# Diagnosing childhood cancer in primary care – a realistic expectation?

**DOI:** 10.1038/sj.bjc.6601733

**Published:** 2004-04-20

**Authors:** R G Feltbower, I J Lewis, S Picton, M Richards, A W Glaser, S E Kinsey, P A McKinney

**Affiliations:** 1 Paediatric Epidemiology Group, Unit of Epidemiology and Health Services Research, University of Leeds, Leeds LS2 9LN, UK; 2 Leeds Teaching Hospitals NHS Trust, Leeds LS9 7TF, UK

**Keywords:** cancer, incidence, health services research, children

## Abstract

The burden of childhood cancer for Primary Care Trusts (PCTs) is unknown. PCTs in Yorkshire are representative of England and Wales and show little heterogeneity in the incidence rates of childhood cancer. Each PCT will expect three to five newly diagnosed children per year. A single GP is likely to see an incident case once every 20 years.

The changing administrative geography of the NHS in England and Wales has resulted in substantial alteration to the ‘organisational’ units involved in the delivery of care (Department of Health, 2001). Each year, in England and Wales, approximately 1300 children aged 0–14 years are diagnosed with childhood cancer (Office for National Statistics, 1999; Welsh Cancer Intelligence and Surveillance Unit, 1999). Each child will live within one of the 301 Primary Care Trusts (PCTs) that are responsible for assessing the health needs and commissioning services for populations within their geographic boundaries.

The National Institute for Clinical Excellence (NICE) in England and Wales is currently developing clinical guidelines for improving the outcomes in childhood and adolescent cancer in collaboration with a designated National Collaborating Centre for Cancer (www.nice.org.uk). The final guidance will include recommendations on diagnostic services and referral guidelines for General Practitioners (GPs) and members of the primary care team.

To contribute new baseline quantitative evidence for NICE and PCT commissioning childhood cancer services, we report the incidence of childhood cancer by PCT, taking Yorkshire as a representative area of England and Wales. We also describe the likelihood of a single GP referring a patient with childhood cancer for treatment.

## MATERIALS AND METHODS

The Yorkshire Specialist Register of Cancer in Children and Young People is a population-based Register which ascertains cases from multiple sources (McKinney *et al*, 1998). Since 1990 more than 90% of subjects' diagnoses have been histologically verified. Details of 1215 children aged under 15 and diagnosed from 1990 to 2001 were used to calculate incidence rates (per million person years) across the two Strategic Health Authorities (SHAs) in Yorkshire. Rates were derived for each PCT (*n*=25) using yearly population data constrained to 2001 local authority mid-year estimates, and compared using the *χ*^2^ test for heterogeneity. The sample size allows us to investigate the number of times each PCT is diagnosed with a particular case of childhood cancer.

Standardised morbidity ratios (SMRs) were also estimated based on the expected numbers for each PCT derived from the overall age- and sex-standardised incidence rate for Yorkshire during the study period. The socioeconomic profile and age and sex distribution for PCTs in Yorkshire were also compared with PCTs from the rest of England and Wales, in order to assess whether our findings could be generalised in other regions of UK.

## RESULTS

The demographic profile of PCTs in Yorkshire is highly representative of England and Wales: the median childhood population counts were 26 700 in Yorkshire compared with 27 400 in the rest of England and Wales, and they ranged in size from 14 800 to 47 400 and 11 400 to 71 600, respectively. The socioeconomic profile in Yorkshire, comparing the median and interquartile range of the Townsend deprivation index, was also similar to the rest of England and Wales.

The incidence rates for all childhood cancers are summarised in Table 1

**Table 1:** Incidence rates for childhood cancer by primary care trust, 1990–2001, ranked in ascending order within SHA

**SHA^a^**	**Primary care trust**	**Population (0–14)**	**Incidence rate^b^**	**95% Confidence interval**
WYORKS	Leeds North East	21,785	110.8	70.4–151.1
	North Kirklees	36,580	119.7	87.4–151.9
	Leeds North West	28,504	123.0	85.8–160.2
	North Bradford	17,855	124.8	77.7–171.8
	East Leeds	35,638	137.4	102.3–172.5
	South Leeds	28,510	141.8	102.1–181.5
	Eastern Wakefield	34,924	142.2	106.2–178.2
	Huddersfield Central	26,818	145.4	103.8–187.0
	Bradford City	35,074	151.5	114.4–188.7
	South Huddersfield	14,638	153.6	95.6–211.7
	Calderdale	38,822	154.0	118.4–189.5
	Airedale	23,462	157.4	110.9–203.9
	Bradford South and West	29,481	160.1	118.5–201.7
	Wakefield West	27,275	166.1	121.8–210.4
	Leeds West	21,912	173.2	123.1–223.3
				
NEYNL	North East Lincolnshire	32,911	113.5	80.3–146.7
	West Hull	26,217	115.9	78.5–153.3
	East Yorkshire	29,403	117.8	81.6–153.9
	Craven, Harrogate and Rural	34,689	135.9	100.2–171.5
	Yorkshire Wolds and Coast	25,219	135.9	93.7–178.1
	Scarborough, Whitby and Ryedale	27,053	138.9	98.3–179.5
	Selby and York	47,303	143.1	111.9–174.3
	Hambleton and Richmondshire	21,473	143.9	97.5–190.3
	Eastern Hull	28,408	147.9	107.3–188.6
	North Lincolnshire	29,717	148.2	107.9–188.5

a Strategic Health Authority: West Yorkshire (WYORKS) and North & East Yorkshire and Northern Lincolnshire (NEYNL).

b Per 1 000 000 person years.

, for each PCT stratified by SHA. The overall incidence rate for Yorkshire was 139.9 per million person years (95% CI 132.0–147.8). Rates in the SHA of West Yorkshire were slightly higher (144.0 (133.5–154.4)) than North East Yorkshire and North Lincolnshire SHA (134.3 (122.3–146.3)). Despite wide variation in incidence for individual PCTs, the differences were not statistically significant (*χ*^2^ test for heterogeneity *P*=0.91) and no significant differences were present between the two SHAs (Mann–Whitney *P*=0.11). This mirrored the situation for all cancers at all ages in Yorkshire between 1995 and 1999 (www.nycris.org.uk/reports/pct9 9/pct99_hyc.pdf).

**Figure 1:**
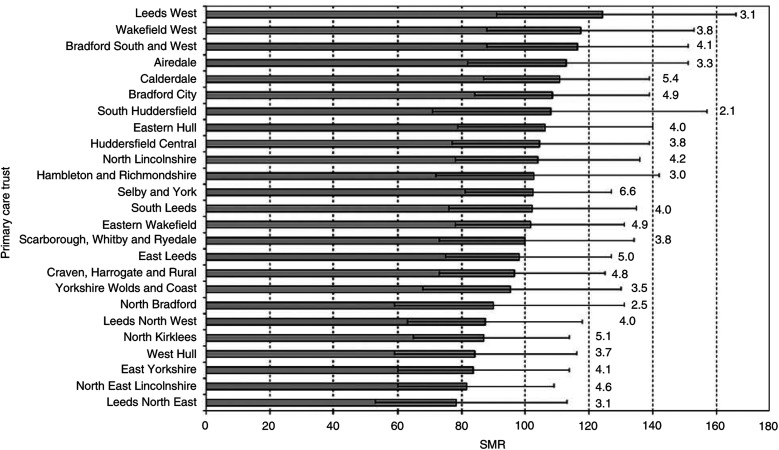
Standardised morbidity ratios and 95% confidence intervals for childhood cancer by primary care trust, 1990–2001. Expected numbers of childhood cancer per year for each PCT, based on the overall age- and sex-standardised incidence rates for Yorkshire.

There was no significant heterogeneity in SMRs across PCTs (*P*=0.90), where the majority of PCTs (84%) could expect three to five resident children to be newly diagnosed with cancer per year (Figure 1

).

Based on the number of registered practitioners defined as unrestricted principals and equivalents (UPEs) during the study period (*n*=2050), a single GP in Yorkshire will see a child diagnosed with cancer once every 20 years on average. The ratio of the number of GPs to population size in Yorkshire was similar to that for the whole of England (approximately 1 : 1750).

## DISCUSSION

These data provide new information on the burden of childhood cancer in PCTs. Our findings relate to new patients for whom 5-year survival will on average be 70%, thus incurring continuing costs for commissioners. Our estimates are derived from a long-term data set, an essential requirement for rare conditions where the smoothing of natural random variation year by year is necessary. However, we have demonstrated that when reliable population-based data are available, healthcare planning can have a quantifiable base. PCT denominator populations are available, but timely geographically matched information on incident cases of less common diseases is not always accessible. The Yorkshire Register routinely collects patient information that is linked to both NHS and census geography, enabling accurate and timely PCT-level information to be published.

Populations served by PCTs vary substantially (Table 1). However, since the data from Yorkshire are representative of England and Wales with respect to PCT population size and socioeconomic circumstance, our findings of an average of 3–5 children per year for each PCT can be generalised for consortia to predict the future numbers of newly diagnosed children. It is more difficult to estimate the longer term overall burden of childhood malignancies due to their diversity, varying treatment regimens and differing prognosis.

Our results have implications for the planning and delivery of healthcare in the primary care setting. They highlight the difficulty for any individual GP being faced with the problem of correctly diagnosing and referring a patient given the likely time lag of 20 years between subsequent consultations. This problem is magnified by the heterogeneity of paediatric malignancies, which differ widely in their pathology and presenting symptoms. For example, typical presenting symptoms will vary for leukaemia, brain tumours and bone tumours. Therefore, it is even more improbable that a GP will see two children with the same malignancy in the course of their career. Currently, we lack any substantive quantitative evidence describing the frequency of GP consultations involving a differential diagnosis for paediatric cancer referrals. To overcome the problem of ‘rarity’ and to ensure that children are rapidly and appropriately referred and diagnosed, specific guidelines for primary care are essential.

Set against this background of an extremely low probability of a single or series of consultations resulting in an eventual diagnosis, Dixon-Woods *et al* (2001) have clearly highlighted a need for GPs to consider and recognise the opinions of parents who insist ‘that there is something wrong’ in the evaluation of the seriousness of a condition. A systems-based approach for GPs might assist them in recognising rare conditions that are difficult to diagnose (Dixon-Woods *et al*, 2001). Health economists and primary care strategists also need to consider carefully where the allocation of funds will most effectively minimise the chances of overlooking a referral of paediatric cancer.

To implement any specific future guidelines effectively, alterations in the current organisation of services for children in primary care may be required, such as the availability of ‘GP paediatricians’. This idea, originally proposed in the 1976 ‘Court Report’, has recently received more attention with increasing numbers of GPs specialising in particular clinical areas (Royal College of GPs and Royal College of Physicians of London, 2001). The model of primary care specialisation, prevalent in some European countries such as Germany, may be of importance in identifying the possible influences on national differences in childhood cancer survival rates (Gatta *et al*, 2003) and demands further consideration.
